# Nomogram model for screening the risk of frailty in older adult atrial fibrillation patients: a cross-sectional study

**DOI:** 10.3389/fpubh.2024.1434244

**Published:** 2024-11-27

**Authors:** Hairong Lin, Mei Lin, Zhiying Xu, Hong Li, Dingce Sun

**Affiliations:** ^1^Department of Gastroenterology, Mianyang Central Hospital, School of Medicine, University of Electronic Science and Technology of China, Mianyang, Sichuan, China; ^2^Department of Nursing, Tianjin Medical University General Hospital, Tianjin, China; ^3^Department of Urology, Mianyang Central Hospital, School of Medicine, University of Electronic Science and Technology of China, Mianyang, Sichuan, China

**Keywords:** frailty, atrial fibrillation, nomogram, sleep disruption, mental health status, chronic diseases

## Abstract

**Background:**

Frailty is common in atrial fibrillation (AF) patients, but the specific risk factors contributing to frailty need further investigation. There is an urgent need for a risk prediction model to identify individuals at high risk of frailty.

**Aims and objectives:**

This cross-sectional study aims to explore the multiple risk factors of frailty in older adult patients with AF and then construct a nomogram model to predict frailty risk.

**Methods:**

We recruited 337 hospitalized patients over the age of 60 (average age: 69, 53.1% male) with AF between November 2021 and August 2022. Data collected included patient demographics, disease characteristics, sleep patterns, mental health status, and frailty measures. We used LASSO and ordinal regression to identify independent risk factors. These factors were then incorporated into a nomogram model to predict frailty risk. The model’s performance was assessed using the concordance index (C-index) and calibration curves.

**Results:**

Among the AF patients, 23.1% were classified as frail and 52.2% as pre-frail. Six risk factors were identified: age, gender, history of coronary heart disease, number of chronic conditions, sleep disruption, and mental health status. The internal validation C-index was 0.821 (95% CI: 0.778–0.864; bias-corrected C-index: 0.795), and the external validation C-index was 0.819 (95% CI: 0.762–0.876; bias-corrected C-index: 0.819), demonstrating strong discriminative ability. Calibration charts for both internal and external validations closely matched the ideal curve, indicating robust predictive performance.

**Conclusion:**

The nomogram developed in this study is a promising and practical tool for assessing frailty risk in AF patients, aiding clinicians in identifying those at high risk.

**Relevance to clinical practice:**

This study demonstrates the utility of a comprehensive predictive model based on frailty risk factors in AF patients, offering clinicians a practical tool for personalized risk assessment and management strategies.

## 1 Introduction

Atrial fibrillation (AF) is the most common arrhythmia, affecting 2 to 4% of adults and approximately 37.57 million individuals worldwide (1). AF significantly heightens the risk of ischemic stroke by 4 to 5 times (2), doubles the likelihood of myocardial infarction (3), and greatly increases the risk of vascular dementia and Alzheimer’s disease (4). Frailty, a geriatric syndrome caused by the decline of physical function and physiological reserves, renders individuals more vulnerable to adverse events and stressors (5). Research indicates that neuroendocrine disorders, chronic inflammation, impaired energy metabolism, social environment, and psychological factors are involved in the occurrence and regression of frailty (6). Given that frailty and AF share similar pathogenic pathways like chronic inflammation and neuromodulation (3) the prevalence of frailty is notably high among AF patients (7). Meanwhile, frailty is strongly linked to increased stroke and bleeding risks in these patients (8), in addition, to reducing the possibility of maintaining sinus rhythm (9). Frailty progresses dynamically and is reversible, thus, early detection of risk factors and targeted interventions can help reverse pre-frailty and slow its advancement. Previous studies have highlighted various sociodemographic and clinical risk factors, covering age, polypharmacy, loneliness, and sleep status (10, 11). However, studies focusing on integrated risk factors for frailty specifically in AF patients are scarce. A prediction model based on these risk factors could more effectively identify high-risk individuals compared to existing diagnostic tools like the FRAIL scale which only provides broad categorizations (12). This model enables more precise and personalized prediction of frailty occurrence. Currently, frailty risk prediction models are widely used in many diseases (13). However, to our knowledge, there has not been a frailty risk predictive tool in the AF field. Among the many tools for visualization of the results of prediction models, the nomogram has simple and intuitive advantages it can quickly and personally calculate risk probabilities (14). Therefore, we aim to construct and validate a nomogram model to predict the risk of frailty in AF patients by combining multi-dimensional risk factors from socio-demographic, behavioral, and mental dimensions, to provide clinicians with a valuable tool to assess frailty risk more accurately in this population.

## 2 Methods

### 2.1 Study participants

This study employed a cross-sectional survey design and included 337 older adult patients diagnosed with AF who were admitted to the Affiliated Hospital of Teaching in Tianjin between November 2021 and August 2022. Among them, 242 patients enrolled from November 2021 to April 2022 were assigned to the training group, while the remaining 95 patients constituted the testing group.

Participants were selected based on the following criteria: (1) a diagnosis of AF according to the European Society of Cardiology guidelines (15); (2) age 60 years and older, in line with Chinese geriatric criteria; (3) ability to communicate effectively and willingness to participate in the study. Exclusion criteria included patients with (1) severe mental illness that would impede cooperation, and (2) reversible AF caused by hyperthyroidism or electrolyte disorders.

### 2.2 Baseline data collection

#### 2.2.1 Baseline data

Baseline characteristics included a range of variables: (1) demographic variables such as age, gender, and education level; (2) clinical parameters including AF type, Body Mass Index (BMI), AF duration, number of chronic ailments, and a history of diabetes, hypertension, coronary heart disease, heart failure, and ischemic stroke; (3) lifestyle factors such as smoking status and alcohol consumption; and (4) laboratory indices comprising low-density lipoprotein (LDL), high-density lipoprotein (HDL), high-sensitivity C-reactive protein (Hs-CRP), brain natriuretic peptide (BNP), left atrial diameter (LA), left ventricular end-diastolic diameter (LVEDD), and left ventricular ejection fraction (LVEF).

#### 2.2.2 Ethics approval and consent

This study received approval from the Ethics Committee for Clinical Research of Tianjin Medical University General Hospital (approval number IRB2022-WZ-053). All procedures adhered to relevant guidelines and regulations. Informed consent was obtained from all participants.

#### 2.2.3 Data collection method

Clinical and biochemical data were retrieved from hospital medical records, while general information was obtained through interviews and questionnaire surveys. Researchers explained the study’s goals and procedures to participants to secure informed consent. Out of 350 distributed questionnaires, 337 were completed and valid, resulting in a high response rate of 96.2%.

### 2.3 Assessment of frailty, mental health status, and sleep status

Frailty was evaluated using the Chinese version of the FRAIL scale, which incorporates five components: fatigue (over the past month), resistance, ambulation (ability to climb stairs or walk 200 meters unassisted), illness (presence of ≥5 chronic conditions), and weight changes (weight loss>3 kg in the past 3 months). Each component is scored between 0 and 1Scores are classified as follows: robust (score of 0), pre-frail (scores of 1–2), and frail (scores of 3–5). The scale demonstrated strong reliability and validity, with a Cronbach’s *α* coefficient of 0.826 (12). Mental health was evaluated using the Mental Health Inventory-5 scale (MHI-5), a validated 5-item subscale of the SF-36 questionnaire which assesses both negative emotions such as anxiety and depression, and positive emotions like happiness and peace experienced over the past month (16). Scores were recorded using a Likert scale from 1 (“All the time”) to 5 (“None”), with total scores ranging from 0 to 100. Previous studies categorized patients into four groups based on their MHI-5 scores: 86–100, 76–85, 53–75, and 0–52, with the86-100 serving as the reference group and scores ≤52 indicating severe depressive symptoms (17). The reliability and applicability of the MHI-5 in AF populations have been extensively demonstrated and utilized (18). Sleep status was assessed across three fronts: sleep duration, sleep disruption, and difficulty falling asleep within the past month. Sleep duration quantifies actual nighttime sleep, sleep disruption tracks the frequency of awakenings not related to nocturia, and difficulty falling asleep assesses the inability to initiate sleep after more than 30 min of preparation.

### 2.4 Statistical analysis

Statistical analysis was performed using SPSS 23.0 and R version 4.1.3. Additional data processing utilized software packages including “MASS,” “Brant,” “RMS,” and “GLMNet.” These tools facilitated comprehensive exploration and interpretation of the collected data. Continuous variables were described using mean ± standard deviation or median (range), while categorical variables were presented as proportions and percentages. Descriptive analysis focused on elucidating the frailty status and socio-demographic characteristics of older adult AF patients. Lasso regression was employed to select variables, addressing multicollinearity and reducing the risk of model overfitting. Variables with a non-zero penalty coefficients were retained as candidates. Ordinal regression was then applied to identify the most significant candidates, which were integrated into a nomogram model for frailty prediction in AF patients. Ultimately, the C-index and the calibration curve were considered to appraise the discrimination and predictive ability of the model, respectively. The C-index is a measure used to assess the discriminative ability of a predictive model, specifically evaluating how well the model can accurately determine the likelihood of an event occurring for a patient. The C-index ranges from 0.5 to 1, where 0.5 indicates that the model’s predictive ability is no better than random guessing, and 1 signifies perfect predictive accuracy. A higher C-index reflects better predictive performance of the model. The C-index>0.7 indicated good discrimination.

The calibration curve is used to evaluate the calibration performance of the predictive model, specifically the consistency between the predicted probabilities and the actual probabilities of the events. Ideally, the calibration curve should be a straight line passing through (0,0) and (1), indicating that the model’s predicted probabilities perfectly align with the actual probabilities. Deviations from this straight line indicate discrepancies between the predicted and actual probabilities. If the curve falls below the line in a certain range, it suggests that the model underestimates the probability of the event occurring in that range; conversely, if the curve is above the line, it indicates that the model overestimates the event probability. *p* < 0.05 was considered to be statistically significant.

## 3 Results

### 3.1 Patient characteristics and baseline comparison

The average age of the patients was 69 ± 6 years. A total 179 were male (53.1%), 30.5% had a smoking history, and 20.7% had a history of alcohol consumption. Among the AF patients, the prevalence rates were 24.7% for robust, 52.2% for pre-frail, and 23.1% for frail individuals (Table 1).

**Table 1:** Basic characteristics of older adult AF patients.

Item	Characteristic	All patients (*n* = 337)	Training group (*n* = 242)	Testing group (*n* = 95)
Frail status	Robust	83 (24.7%)	55 (22.7%)	28 (29.5%)
	Pre-frail	176 (52.2%)	130 (53.7%)	46 (48.4%)
	Frail	78 (23.1%)	57 (23.6%)	21 (22.1%)
Age (year)	—	69 ± 6	69 ± 6	68 ± 7
BMI (Kg/m^2^)	—	25.56 ± 3.38	25.8 ± 3.4	24.85 ± 2.91
Duration (month)	—	20 (7.65)	29 (9.77)	12 (3.36)
Number of chronic diseases	—	3.68 ± 1.79	3.83 ± 1.78	3.32 ± 1.78
Gender	Male	179 (53.1%)	135 (55.8%)	44 (46.3%)
	Female	158 (46.9%)	107 (44.2%)	51 (53.7%)
AF type	Paroxysmal	208 (61.7%)	150 (62.0%)	58 (61.1%)
	Persistent	129 (38.3%)	92 (38.0%)	37 (38.9%)
Education	Primary school	49 (14.5%)	35 (14.5%)	14 (14.7%)
	Junior high school	118 (35.0%)	89 (36.8%)	29 (30.5%)
	Senior high school	92 (27.3%)	63 (26.0%)	29 (30.5%)
	Junior college	78 (30.2%)	55 (22.7%)	23 (24.2%)
Diabetes	Yes	73 (21.7%)	48 (19.8%)	25 (26.3%)
Hypertension	Yes	217 (64.4%)	166 (68.6%)	51 (53.7%)
Coronary heart disease	Yes	133 (39.5%)	93 (38.4%)	40 (42.1%)
Heart failure	Yes	37 (11.0%)	18 (7.4%)	19 (20.0%)
Ischemic stroke	Yes	88 (26.1%)	60 (24.8%)	28 (29.5%)
Smoking	No	234 (69.4%)	165 (68.1%)	69 (72.6%)
	Smoking cessation	51 (15.1%)	42 (17.4%)	9 (9.5%)
	Yes	52 (15.4%)	35 (14.5%)	17 (17.9%)
Drinking	No	267 (79.2%)	184 (76.0%)	83 (87.4%)
	Abstinence	23 (6.8%)	22 (9.1%)	1 (1.1%)
	Yes	47 (13.9%)	36 (14.9%)	11 (11.6%)
Sleep duration	>7 h	97 (28.8%)	71 (29.3%)	26 (27.4%)
	5–7 h	195 (57.9%)	137 (56.7%)	58 (61.1%)
	<5 h	45 (1345%)	34 (14.0%)	11 (11.6%)
Sleep disruption	None	102 (30.3%)	67 (27.7%)	35 (36.8%)
	<3 times/week	81 (24.1%)	125 (51.7%)	23 (24.2%)
	≥3 times/week	154 (45.7%)	117 (48.3%)	37 (38.9%)
Difficulty falling asleep	No	132 (39.2%)	85 (35.1%)	47 (49.5%)
	Yes	205 (60.8%)	157 (64.9%)	48 (50.5%)
LDL (mmol/L)	–	2.68 ± 0.86	2.66 ± 0.89	2.74 ± 0.79
HDL (mmol/L)	–	1.12 (1.01, 1.30)	1.12 (1.02, 1.29)	1.13 (0.96, 1.35)
Hs-crp (mg/L)	–	1.42 (0.75, 2.80)	1.42 (0.73, 2.54)	1.43 (0.82, 3.67)
BNP (pg/ml)	–	143 (71,282)	139 (70,273)	164 (76,314)
LA (mm)	–	42.21 ± 5.55	42.11 ± 5.43	42.48 ± 5.90
LVEDD (mm)	–	48.08 ± 4.16	48.08 ± 4.03	48.06 ± 4.53
LVEF (%)	–	62 (60.63)	62 (60.63)	62 (59.63)
Mental health status (points)	–	74.60 ± 16.62	75.68 ± 15.62	72.75 ± 16.87

Basic data are expressed as Mean ± SD or Median (IQR) or Number (%). *p < 0.05. LA, left atrial diameter; LV, left ventricular end-diastolic diameter; LVEF, Left ventricular ejection fraction; BMI, body mass index; LDL, low-density lipoprotein; HDL, high-density lipoprotein; Hs-crp, high-sensitivity c-reactive protein; BNP, brain natriuretic peptide, troponin.

### 3.2 Screening variables

**Figure 1:**
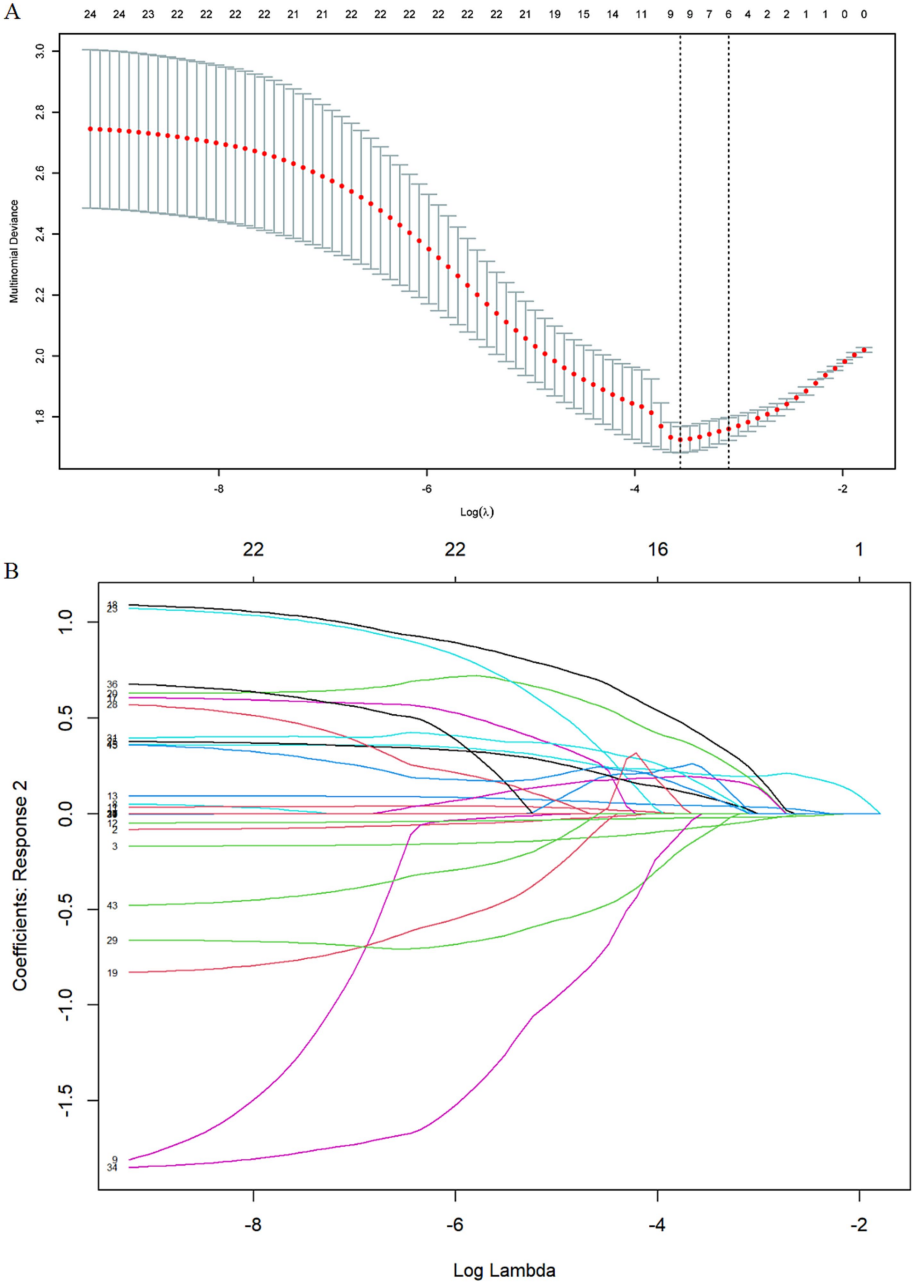
Variables selection by using least absolute shrinkage and LASSO regression. (A) Cross-validation plot for the penalty term: The 2 dashed lines correspond to two special lambda values: lambda. Min(left) and lambda. l SE (right). We ultimately selected the six variables associated with the lambda.1 SE value. (B) Values of the penalty parameter: The curve in the figure represents the change trajectory of each independent variable coefficient, the vertical coordinate is the value of the corresponding coefficient of the independent variable, the lower abscissa is log(*λ*), and the upper abscissa is the number of variables with non-zero coefficients in the model at this time.

Variable selection utilized lasso regression with fourfold cross-validation, identifying six key predictors from an initial pool of 25 variables. The selected predictors included age, gender, history of coronary heart disease, number of chronic diseases, sleep disruption frequency, and mental health status (Figure 1). The parallel line test confirmed the suitability of ordinal regression with a. *p*-value of 0.69. The ordinal regression results revealed significant associations with frailty for the following predictors: age (66–70 years: OR, 1.0 [95% CI, 0.51–1.92]; 71–75 years: OR, 2.28 [95% CI, 1.02–5.19]; 76–80 years: OR, 3.91 [95% CI, 1.43–15.86]; ≥80 years: OR, 4.13 [95% CI, 1.14–15.86]; *p* = 0.005), gender (female: OR, 1.86 [95% CI, 1.06–3.31]; *p* = 0.029), history of coronary heart disease (OR, 2.46 [95% CI, 1.31–4.67]; *p* = 0.004), number of chronic diseases (>4: OR, 5.08 [95% CI, 2.57–10.36]; *P* < 0.001), sleep disruption <3 times/week: OR, 1.50 [95% CI, 0.71–3.19]; ≥3 times/week: OR, 2.59 [95% CI, 1.36–4.98]; *p* = 0.012), and mental health status (76–85 points: OR, 1.45 [95% CI, 0.74–2.86]; 53–75 points: OR, 3.00 [95% CI, 1.39–6.57]; 0–52 points: OR, 6.98 [95% CI, 2.40–21.30]; *P* < 0.001) were significantly associated with frailty (Table 2).

**Table 2:** Ordinal regression analysis of frailty in older adult AF patients.

Item	*P*	*OR*	OR (95%CI)	
			Lower limit	Upper limit
Age				
60–65	0.005*	–	–	–
66–70		1	0.51	1.92
71–75		2.28	1.02	5.19
76–80		3.91	1.43	15.86
≥80		4.13	1.14	15.86
Gender				
Male	0.029*	–	–	–
Female		1.86	1.06	3.31
History of coronary heart disease				
No	0.004*	–	–	–
Yes		2.46	1.31	4.67
Number of chronic diseases				
≤4	<0.001*	–	–	–
>4		5.08	2.57	10.36
Sleep disruption				
No	0.012*	–	–	–
<3 times/week		1.5	0.71	3.19
≥ 3 times/week		2.59	1.36	4.98
MHI-5 score				
86–100	<0.001*	–	–	–
76–85		1.45	0.74	2.86
53–75		3	1.39	6.57
0–52		6.98	2.4	21.3

*P < 0.05. OR, odds ratio.

### 3.3 Development of a nomogram model for frailty prediction in AF patients

**Figure 2:**
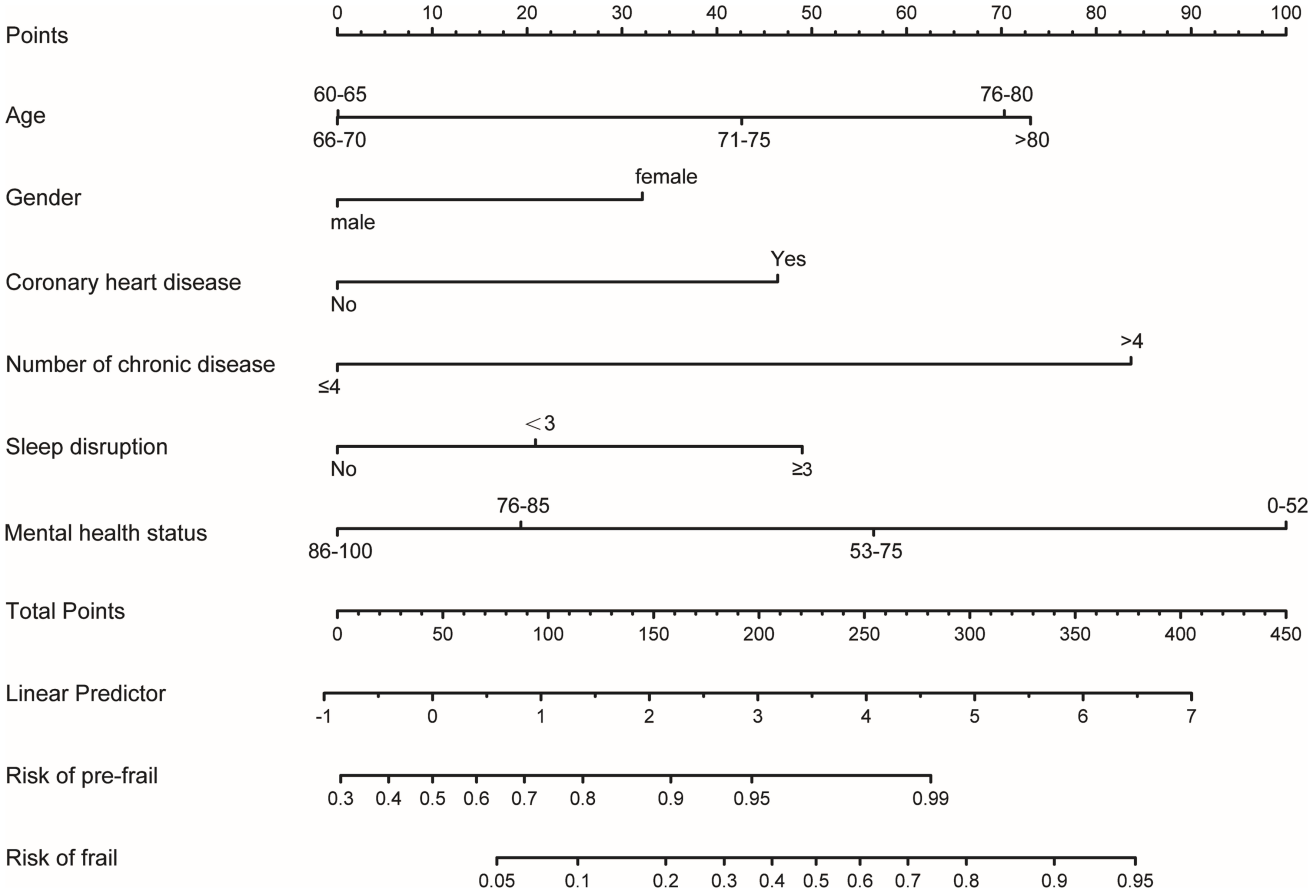
Nomogram for estimating frailty probability in older adult patients with AF. This nomogram includes age, gender, number of chronic diseases, history of coronary heart disease, sleep disruption, and mental health status. The horizontal scale labeled “Points” reflects the impact of each variable. Draw a line up to the points axis for each variable, The total score was calculated by summing all the variables. Then, the probability of pre-frail and frail was acquired by drawing a line down from the total points axis to the horizontal axis “Risk of pre-frail” and “Risk of frail” below.

Based on the ordinal regression results, we developed a nomogram model to predict frailty risk among AF patients (Figure 2). Each predictor in the nomogram is assigned a specific score displayed at the top. Clinicians can calculate a patient’s total score by summing these values and then estimate the probability of pre-frailty and frailty by drawing a line from the total score to the risk axis. For instance, a 74-year-old male with a history of coronary heart disease, a mental health status score of 52, more than four chronic diseases, and sleep disruption exceeding three times per week, would have a total score of 323.5, indicating a 0.99% risk for pre-frailty and an 85% risk for frailty.

### 3.4 Performance of the nomogram model

**Figure 3:**
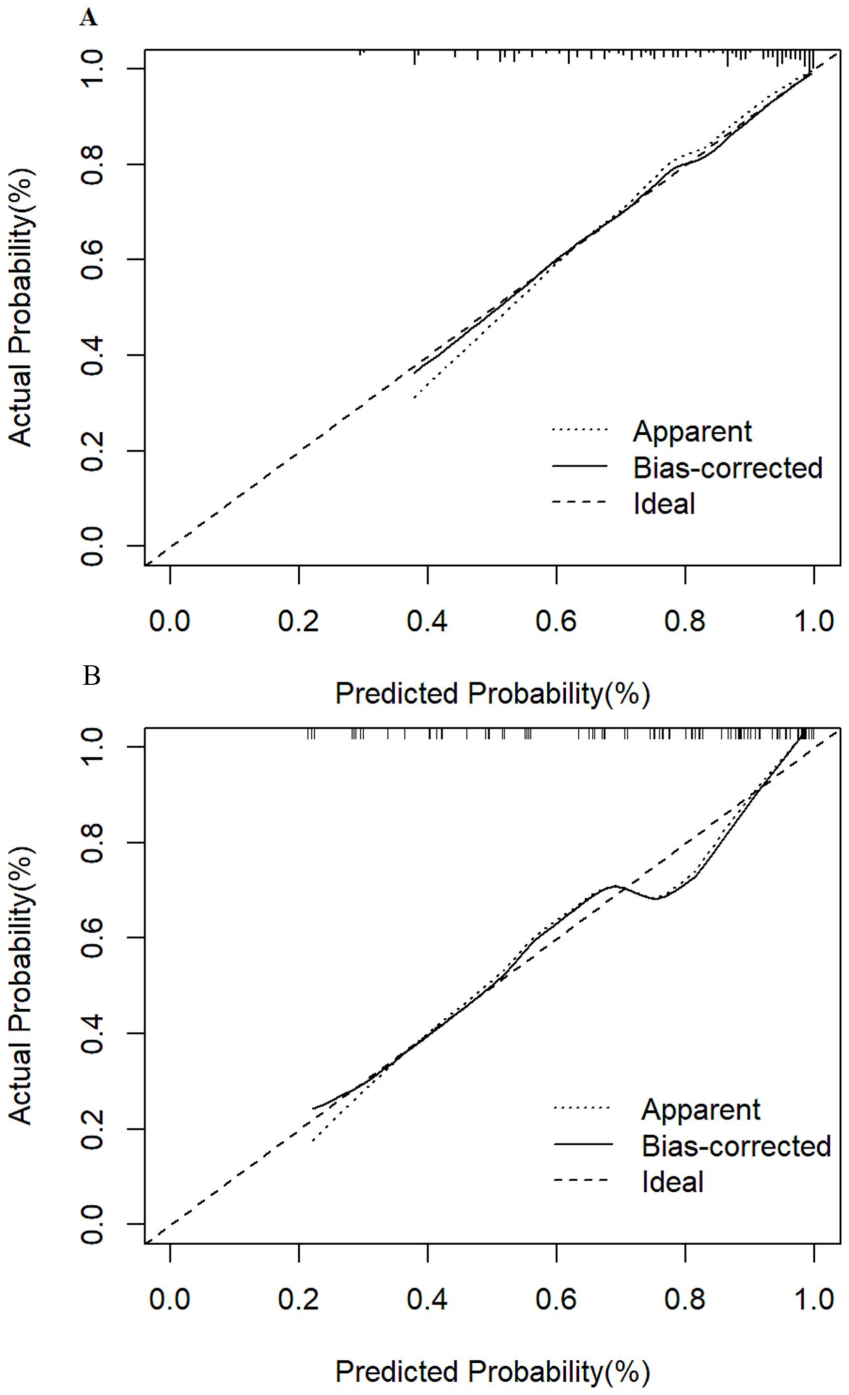
Calibration curves for frailty risk prediction model. (A) Calibration curves of the training group show that the apparent curve closely matches the ideal curve, indicating excellent predictive probability. (B) Calibration curves of the testing group also demonstrate that the apparent curve aligns well with the ideal curve, confirming the model’s strong predictive performance.

The nomogram model’s performance was assessed through discrimination and calibration plots. Calibration plots (Figure 3) demonstrated a high degree of alignment between actual and ideal curves, reflecting strong predictive accuracy. The C-index for the training group (0.821, 95% CI: 0.778–0.864; bias-corrected C-index: 0.795) and testing group (0.819, 95% CI: 0.762–0.876; bias-corrected C-index: 0.819) underscored the model’s strong discriminatory power.

## 4 Discussion

### 4.1 Improved risk prediction with multidimensional predictors

The study developed and validated a nomogram model to predict the risk of frailty in AF patients. Key predictors included age, gender, history of coronary heart disease, number of chronic illnesses, sleep disturbances, and mental health status emerged as independent predictors. Both internal and external validations consistently affirmed the model’s robust discriminatory and calibration capabilities. The prevalence of pre-frailty and frailty in our AF cohort were 52.2 and 23.1%, respectively—aligned closely with meta-analytic findings (pre-frailty: 39.7%, range: 29.9–50.5%; frailty: 35.0%, range: 26.1–45.1%) (19). Comparatively, the prevalence of frailty in Chinese community residents is lower, reported at 9.9%, with a range of 2.3 to 12.7% (20). The higher frailty rates in the AF cohort could be attributed to shared underlying pathogenic mechanisms (3). Frail AF patients exhibited elevated risks of all-cause mortality, ischemic stroke, and bleeding (19). Hence, identifying risk factors and constructing predictive models are imperative for assessing frailty risk in the AF population. Prior studies enrolled diversiform factors such as dietary habits, age, exercise habits, and social support into frailty risk models, which demonstrated that incorporating comprehensive predictors is more effective than relying solely on physiological indicators, given that frailty results from multi-systems working together (21).

Our study’s innovation lies in integrating diverse risk factors encompassing demographic, sociological, lifestyle, mental health, and sleep-related parameters. This nomogram model is user-friendly, enabling clinicians to swiftly compute patient frailty risks with intuitive ease.

### 4.2 AF patients with advanced age, female, and various chronic diseases are more prone to frailty

Aging leads to differential declines in physiological systems, notably marked changes in skeletal muscle. Firstly, there is a reduction in muscle contractile tissue and an increase in non-contractile tissue, such as fat and connective tissues (22). Secondly, skeletal muscle experiences a decrease in capillary density and oxidative capacity (23). Even with high-protein diets or physical exercise, muscle protein synthesis rates decline (24). Concurrently, degeneration of the basal ganglia affects motor planning, thereby compromising motor control (22). These changes contribute to decreased muscle quantity and mass, culminating in reduced muscle strength (24). Physical activity has been shown to enhance muscle strength and attenuate frailty progression (25). However, the relationship between AF and exercise is nuanced; long-term endurance exercise may increase AF risk in a J-shaped pattern, while mild to moderate physical activity provides protection against AF (26). Gender differences exist in the association between exercise and AF; moderate to vigorous exercise reduces AF risk in women, whereas vigorous exercise increases risk in men (27). Clinicians should tailor exercise recommendations to the type, intensity, and duration of activity and address psychological barriers like kinesiophobia that hinder physical activity (28). Older adult AF patients are particularly vulnerable to multimorbidity including heart failure, stroke, and coronary artery disease. In our study, 30.2% of older adult AF patients exhibited four or more concurrent diseases, exacerbating frailty progression under chronic stress. Notably, AF patients with coronary artery disease demonstrated a 2.4-fold higher frailty risk.

The challenge of managing multiple chronic conditions often leads to polypharmacy, which increases the risk of adverse drug effects and, consequently, frailty (29). Frailty itself also increases the risk of drug-related harm (30). Thus, careful medication management, including appropriate dosages and schedules, is essential for older adult AF patients.

Furthermore, we identified that women had a 1.86-fold higher frailty risk compared to men. Older women exhibit lower skeletal muscle mass and higher fat mass relative to older men (31), partly due to postmenopausal estrogen depletion (32). Cultural and lifestyle choices, such as engaging in high-intensity household activities without sufficient structured exercise, may also contribute to functional impairments and frailty progression among older women, particularly in Chinese populations (33).

### 4.3 AF patients with sleep disruption are more prone to frailty

Studies indicate a high prevalence of sleep issues among AF patients (34). Our research demonstrates a positive correlation between sleep disruption and increased frailty, possibly exacerbated by the symptom burden associated with AF (35). During the night, with the activation of the vagus nerve, the incidence of symptoms such as palpitations and dyspnea increases. In addition, reduced sensory stimulation from the environment leads patients to be more sensitive to symptoms. These effects stack up, leading to an increased risk of sleep disruption. Sleep disruption causes dysfunction of the hypothalamic–pituitary–adrenal axis and gonadal axis, decreased cortisol responsiveness, and decreased levels of growth hormone and insulin-like growth factor-1 (36), which are crucial in frailty development. Another study noted a significant association between sleep duration and frailty (37). However, this study did not obtain the same effect which may be related to only considering the night sleep time and ignoring the factors like napping. To mitigate these effects, healthcare providers should advise patients to reduce electronic device use before bedtime, use relaxing music, and optimize their sleep environment.

### 4.4 AF patients with negative mental health status are more prone to frailty

Our investigation underscores a negative correlation between mental health status and frailty in AF patients. Those scoring ≤52 on the MHI-5 scale faced a 6.4-fold higher frailty risk, echoing findings by Uchmanowicz I (38). Negative mental states trigger neuroimmune responses that increase inflammatory cytokine release, leading to muscle mass and strength decline and thus promoting frailty. These inflammatory processes additionally impact brain regions managing emotions like fear and anxiety, exacerbating conditions such as anxiety and depression (39). A meta-analysis confirmed that depression increased frailty prevalence by fourfold, while frailty also significantly raised depression incidence. (40). Clinical strategies should prioritize assessing mental health in older adult AF patients and recommending interventions such as aromatherapy and meditation for emotional stabilization.

### 4.5 Strengths and limitations

This study offers valuable insights into the factors influencing frailty in AF patients and has developed a predictive model for frailty risk. This model enables healthcare providers to assess frailty risk more accurately. However, the study has limitations, including a small sample size and the absence of large-scale multicenter trials. Furthermore, frailty was assessed using subjective Frail scales rather than objective measures such as grip strength and stride length. The focus on hospitalized patients also introduces potential selection bias.

## 5 Conclusion

Frailty emerges as a prevalent condition among older adult patients with AF. Factors such as age, gender, history of coronary heart disease, comorbidity burden, sleep disturbances, and mental health status significantly influence frailty development in AF patients. A nomogram model incorporating these significant risk factors demonstrates robust predictive and discriminative capabilities.

## Data Availability

The data that support the findings of this study are available on request from the corresponding author.
